# Outcome of selective non-diverting low anterior resection after neoadjuvant chemoradiotherapy and curative surgery for proximal rectal cancer: A prospective case series

**DOI:** 10.34172/mejdd.2024.396

**Published:** 2024-10-30

**Authors:** Aidin Yaghoobi Notash, Ehsan Sadeghian, Ehsan Sobhanian, Behnam Behboudi, Seyed Mohsen Ahmadi Tafti, Zahra Moghimi, Amir Keshvari, Mohammad Sadegh Fazeli, Mohammad Reza Keramati

**Affiliations:** ^1^Tehran University of Medical Sciences, Tehran, Iran; ^2^Department of Surgery, Shariati Hospital, Tehran University of Medical Sciences, Tehran, Iran; ^3^Department of Surgery, Sina Hospital, Tehran University of Medical Sciences, Tehran, Iran; ^4^Department of Colorectal Surgery, Colorectal Research Center, Imam Khomeini Hospital Complex, Tehran University of Medical Sciences, Tehran, Iran; ^5^Department of Gynecology, Yas Hospital, Tehran University of Medical Sciences, Tehran, Iran

**Keywords:** Low anterior resection, Defunctioning stoma, Anastomotic leakage, Defunctioning loop stoma

## Abstract

**Background:**

Low anterior resection (LAR) is the gold standard for curative cancer treatment in the middle and upper rectum. In radically operated patients, the local recurrence rates with total mesorectal excision (TME) after 5 and 10 years was<10%, with 80% in 5 years survival. Anastomotic leakage (AL) affects 4%–20% of patients who underwent LAR. Based on some studies, there is a risk reduction of symptomatic AL after LAR and the need for reoperation in patients with a defunctioning stoma (DS), also known as diverting stoma. Ileostomy has many complications, such as skin irritation and leakage, dehydration, obstruction, and parastomal hernia. Considering the complications of defunctioning loop-ileostomy (DLI) we designed this study to evaluate noninserting stoma in a particular group of patients.

**Methods:**

This retrospective cohort case series study utilized data of 20 patients with rectal adenocarcinoma with lesion>7 cm from anal verge in rectoscopy who underwent LAR after 28 sessions of chemoradiotherapy (CRT) and 6 weeks of rehabilitation. All of the patients matched our criteria, so DLI was not performed on any of them.

**Results:**

Among our 20 patients, four AL were happened (20%). C-reactive protein (CRP) on post-operation day (POD) six was valuable. Computed tomography (CT) scan was not used as a reliable modality in our study. In all patients with positive AL, magnetic resonance imaging (MRI) was useful and reported correctly, and direct vision of the anastomosis site by rigid rectoscopy was not safe enough to make decisions about it.

**Conclusion:**

The leakage rate was not far from the average leakage rate in other studies. Then it seems it is possible to forget about defunctioning loop stoma (DLS) in safe cases to reduce the stoma complications. Due to our restricted case selection and our close observation protocol, we had no significant complications compared to other studies. According to this study, not inserting stoma in suitable cases with restricted protocol selection is possible, and the leakage rate is not higher in comparison with patients with stoma.

## Introduction

 Low anterior resection (LAR) is known as the gold standard for the curative treatment of cancer in the middle and upper rectum.^1^ This method helps anal sphincter saving without compromising the oncological outcome anymore.^2^ After the introduction of total mesorectal excision (TME), a major advance was made in the surgical strategy for rectal cancer, which resulted in a reduction of local recurrence without adjuvant therapy. In radically operated patients, the local recurrence rates with TME after 5 and 10 years was < 10%, with 80% in 5 years survival.^3-6^ Anastomotic leakage (AL) is a kind of nightmare complication for every surgeon, affecting 4%–20% of patients who underwent LAR.^7,8^ There are several risk factors known for AL; some of them are male sex, smoking, excess alcohol use, overweight, advanced *American Society of Anesthesiologists (ASA)* class, diabetes mellitus, renal, vascular diseases, tumor size, neoadjuvant therapy, anastomotic height from the anal verge and absence of a defunctioning stoma (DS). Some studies have shown a risk reduction of symptomatic AL after LAR and the need for reoperation in patients with a DS, also known as diverting stoma.^8-17^ DS is more preferred to be used as a defunctioning loop-ileostomy (DLI) and, in rare cases, as a loop colostomy.^18,19^ The possibility of becoming a DLI to permanent stoma is about 25% of the cases. There is a wide range of local and systemic physiological changes due to DLI, from skin irritation and leakage (59%) to more important complications such as dehydration, obstruction, and parastomal hernia (25%).^20^ One-third of DLI patients are at risk for dehydration in the first 6 weeks, and half of them need to be admitted for electrolyte correction, which can take their adjuvant chemotherapy at delayed risk. DLI also can affect health and quality of life.^21,22^ Some surgeons believe in routine fecal diversion. They think it results in a lower incidence of AL and the need for surgical intervention. While another group thinks there are similar leakage and mortality rates despite the presence of a stoma. The second group recommends a selective approach, with stoma formation only when there are some concerns about the anastomosis, like incomplete doughnuts or a positive air leak test.^2^

 We are going to evaluate the results of LAR surgery without DLI in a group of patients with the same risk of AL, who have a successful operation and have safe anastomosis in the sight of the surgeon. The next aim is to see the value of some parameters for early AL detection and secondary DLI placement before the occurrence of fatal leak complications.

## Materials and Methods

###  Study Design

 This retrospective cohort case series study utilized data from the Tehran Surgery Research Center, known for its high validity and comprehensive coverage of patients with rectal cancer in Iran. The study focused on patients who underwent rectal cancer surgery, LAR after 28 sessions of chemoradiotherapy (CRT) and 6 weeks of rehabilitation within 2021-2022. The cancer pathologies were adenocarcinoma, and we selected the patients whose lesions were > 7 cm from the anal verge in rectoscopy. Data were analyzed using SPSS software (version 27.0.1 IF026; IBM Corp, Armonk, NY, USA). statistical analysis was conducted using independent samples *t* test. When required, the chi-squared analysis was applied. Statistical significance was set at a *P* value of <0.05 for all analyses.

###  Patient Selection

 Our study was conducted among 20 patients with approved rectal adenocarcinoma in their colonoscopy. All of the patients had 26 sessions of CRT and were scheduled for the operation with 6 weeks delay. The operations started with a mid-line incision on the abdomen. Before starting any procedure, we had an evaluation for existing intra-abdominal metastasis, and tumor local circumstances were also examined. All 20 patients seemed operable at first look. Then LAR started with a TME plan, followed by complete mobilization of the descending colon and left flexure. After exploring and highlighting the inferior mesenteric artery and vein, lymph node dissection and resection were performed as well. After complete tumor resection with 5 cm margin from the tumor edges, anastomosis was performed with Ethicon and Covidien staplers, and all of the anastomoses were checked for complete integrity with air insufflation underwater, and none of the patients had any signs of primary AL. Drainage was performed in all patients.

 All 20 operations were performed by a single experienced colorectal surgeon in our center from the beginning to the end, and all computed tomography (CT) scans and magnetic resonance imaging (MRI) were reported by a single experienced abdominal radiologist, and the ALs were confirmed by a second radiologist independently.

###  Inclusion Criteria

1. Patients with adenocarcinoma of the rectum who underwent 28 sessions of CRT with 6-week delay for the rehabilitation period and are the candidates for LAR.
2. Stable conditions in the surgeon’s opinion about the circumstances during surgery, including properly preoperative mechanical bowel preparation, absence of bowel obstruction, no emergency surgery, safe anastomosis, no massive bleeding during surgery, normal lab data before surgery, no other underlying diseases and no significant drug history like corticosteroids, insulin, etc.
3. Tumoral lesions > 7 cm distance from the anal verge.
4. In all patients, we used standard and similar staplers, including:
  - 29 Covidien annular staplers for the rectosigmoid anastomosis
  - Green Ethicon contour for rectal stump
5. All patients had their CRT in our center with the same and safe methods and had no significant complications within the therapy.
6. Only patients which are at the stage: T3N1

###  Excluding Criteria

1. Patients without CRT
2. Patients with non-treated immunocompromised disease (e.g, corticosteroids, uncontrolled diabetes mellitus, systemic lupus erythematous, etc)
3. Patients with ASA scores of 3 and 4.
4. Patients with any kind of complications that make the surgeon to loop diverting stoma insertion like unsafe anastomosis, massive hemorrhage during surgery, …
5. Microscopically non-radical resections
6. Patients with more than T3N2M0 in the primary evaluation

### Data Collection (Lab Data, Radiological, Clinical, and Paraclinical Data)

- Complete blood count (CBC) diff: Daily check from the third postoperative day to 7^th^
- *Erythrocyte sedimentation rate* (ESR) and CRP: Check in 4^th^ and 5^th^ days postoperatively
- Abdominopelvic CT scan with intravenous (IV) contrast: between days 5-7 postoperatively
- Abdominopelvic MRI with IV contrast: Between days 5-7 postoperatively
- If there were any signs of AL in the CT scan and MRI, patients were scheduled for rigid sigmoidoscopy, and if there were not any signs of AL (healthy anastomosis), patients were dismissed from hospital on day 7 postoperatively.

In patients with positive leakage, based on our practice definition, we considered reoperation, including laparotomy, peritoneal irrigation, and loop diversion stoma insertion. Patients were visited weekly in the clinic after discharge from the hospital. In patients with clinic follow-up, if there were any signs of AL, CBC diff, CT Scan, and MRI were reconsidered, and if AL approved, they had to be planned for delayed stoma insertion.

 We considered CT scan and MRI both due to lack of statistical superiority in available sources and also because of the mortality rate of missed patients with AL.

###  AL Definitions

- Radiological leakage: (All radiological examinations were reported by one individual expert radiologist)

1. Any abscess formation near anastomosis
2. Air exiting out of luminal bowel

- Clinical leakage:

1.  > 38-degree centigrade fever and any symptoms of peritoneal irritation
2. Leukocytosis for more than 72 hours
3. Fecal or pus secretion from the abdominal drain
4. Peritoneal pain and pus secretion from the anus
5. CRP > 135 on day 5 postoperatively

AL was considered below finally:

- Unexplained clinical symptoms of leakage or other complications were checked with a rectoscope if there were any suspicion remained
- Radiological symptoms of leakage were confirmed by two radiologists and accompanied by at least one clinical criteria and checked by rectoscope if there were any suspicions remained

###  Multidisciplinary Assessment

 All patients included in this study underwent a comprehensive assessment by a multidisciplinary team conference, ensuring that treatment decisions were made collaboratively and following the established clinical guidelines.

## Results

 Twenty patients in our study with rectal adenocarcinoma were prepared for LAR. The mean age was 62.7, with 15 (75%) female and five (25%) male. Among them, four patients had reoperation due to AL (20%), and all of them were female (*P* = 0.887). Average CRP on 4^th^, 5^th^, and 6^th^ postoperative days was 138, 138, 79 in the reoperation group, respectively, and 96, 85, 64 in the other group with the *P* value of 0.072, 0.185, 0.042in each day. Among four patients who had AL, we performed a CT scan in three patients (we did not perform a CT scan in one patient because of clinical condition and for prevention of any time loss). Overall, we had four positive leakage reports on the CT scan: two patients with true AL and two patients without any leakage. Actually, two other patients with positive leakage were missed and had no signs of leakage in the CT Scan (*P* = 0.097). Three patients with AL had positive MRI signs of leakage, and one patient had a negative report of leakage. A positive leakage was reported in one patient who had no AL. Therefore, there was one patient in each group with a false report in MRI (*P* = 0.013). Finally, we performed a rectoscopy on all patients. Surprisingly, abnormal rectoscopy was reported in 15 patients: four patients with true leakage and 11 patients without leakage. As we can see, rectoscopy is a diagnosis modality with a high false positivity rate (*P* = 0.53).

 Bowel ileus happened in five patients, three patients with AL (75%), and two patients with no AL (*P* = 0.032).The average day of ileus was 4-6 days postoperatively.

## Discussion

 AL after LAR is an important fatal complication that needs rapid diagnosis and action as well to prevent mortality. To achieve a new way of treating rectal cancer, it is necessary to consider all of the available modalities and evaluate their efficiency.

 Mrak and colleagues in 2016 had a comparison study between two groups of patients with rectal cancer; one group did not install any DLS, and the other one had DLS in a randomized process. In that study, patients with DLS significantly had a lower AL. They indicated that the operation rate for leakage was significantly lower in patients with stoma compared with patients without stoma. They concluded that a protective stoma and the female sex were the only parameters significantly associated with a decreased leakage rate.^23^ While all of our leakages happened in females, two out of 72 patients who had no stoma underwent reoperation due to leakage and bowel obstruction. In our study, five patients were diagnosed with bowel obstruction; three of them had leakage out of four leakages, and two patients had bowel obstruction without any leakage between 16 cases. Our statistical analysis shows the *P* = 0.032in obstruction which indicates the value and importance of bowel obstruction as a red flag for leakage.

 In Tan and colleagues’ study, which is a kind of meta-analysis, 21 studies were included; in 15 studies, leakage rates were lower with stoma, four studies had similar rates, and two studies reported a higher leakage rate with stoma. The meta-analysis reported a significantly lower risk of leakage in patients with stoma.^2^

 The newest study was published by Munshi et al,^24^ and it was conducted in two periods of time (2007-2009 and 2016-2018) among 3948 patients with rectal cancer who performed with LAR. The leakage reported in this study showed no significant difference between with or without DLI. This study introduced male sex, ASA class 3-4, BMI > 30, and neo-adjuvant therapy as the risk factors of leakage. In the study of Talboom et al^25^ which was conducted among 125 patients, 44 patients underwent CT scans, and it was highly suspicious for leakage in 23, with confirmed reports in all those with true leakage and was false negative in one patient (sensitivity 96%).

 Based on the study (a systematic review) of Singh et al in 2014, CRP cut-off values for AL were 172 mg/L on postoperative day (POD) 3, 124 mg/L on POD 4 and 144 mg/L on POD 5.^26^

 In another recent study published in 2022 by Talboom et al, AL rate was 55%, and they evaluated the value of CT scan in the diagnosis of AL. Out of 125 patients with rectal cancer, 44 patients underwent a diagnostic CT scan for suspicion of AL. Eventually, AL was confirmed in 23 of 24. In other patients with no leakage, CT reports pointed to no or minor suspicion of leakage (specificity of 100%).^25^

 Yu et al have studied MRI accuracy in patients with rectal cancer and AL. The overall sensitivity and specificity were 94% and 80%, respectively. They reported T2 weighted imaging could effectively reveal the leak track.^27^

 We designed a case series study with robust screening limitations, and we reported the results in 20 patients. We included only patients with the stage of T3N1, and the anastomosis height from the anal verge was > 7 cm in all cases. The leakage rate was 20%, which is not far from the average leakage rate in other studies. Then, it seems it is possible to forget about DLS in safe cases to reduce the stoma complications itself, and due to our restricted case selection and our close observation protocol, we had no significant complication in comparison to other studies. The most important point is to make sure that it is possible to not insert stomas in properly selected patients because the complications and leakage rate are not more in comparison with patients with stomas, and if any complication or leakage happens, it is conceivable to inhibit mortality and make the best decision at the right time.

 Based on our study, paraclinic and laboratory studies like CRP on POD 6 and MRI on day 4-6 have valuable information that can help us to detect any AL along with physical examinations. CT scan was not reliable in our study, but we would not suggest ignoring information about it due to our limited case numbers.

 Direct vision of anastomosis is not recommended based on our experience due to false positive results. The criterion of AL in rectoscopy is to see fibrins at the site of anastomosis. This finding can be useful after 2 weeks, and before that time, it is not related to the leakage completely. Perhaps this is the reason for the high false negative rate in rectoscopy.
